# How often is patent foramen ovale an innocent bystander?

**DOI:** 10.1002/ccr3.1237

**Published:** 2017-10-26

**Authors:** Francesco Versaci, Giampiero Vizzari, Domenico Sergi, Giuseppe Andò, Antonio Trivisonno, Francesco Romeo

**Affiliations:** ^1^ Department of Cardiovascular Disease Tor Vergata University of Rome Rome Italy; ^2^ Department of Clinical and Experimental Medicine University of Messina Messina Italy; ^3^ Department of Cardiovascular Disease “Antonio Cardarelli” Hospital Campobasso Italy

**Keywords:** Brugada syndrome, palpitations, patent foramen ovale, PFO closure, presyncope

## Abstract

Patent foramen ovale (PFO) is a risk factor for cryptogenetic stroke; its closure should be considered in selected patients. It is not always clear whether symptoms (presyncope, paresthesia) apparently due to paradoxical embolism are related with other cardiovascular disorders such as arrhythmias. Flecainide administration for post‐PFO‐closure supraventricular arrhythmias can unmask a latent undiagnosed Brugada syndrome.

## Introduction

Patent foramen ovale (PFO) is present in about one‐quarter of the adult population, and it has been implicated as a risk factor for cryptogenetic stroke (CS), with a mechanism likely consisting in paradoxical embolism 1, 2. Recent guidance from the American Heart Association and the American Stroke Association Council on Stroke suggests that PFO closure using dedicated devices could be a therapeutic approach in specific cases 3.

Unfortunately, in patients with PFO, it is not always clear whether symptoms, mostly nonspecific, are clearly consistent with a cryptogenetic stroke/transient ischemic attack (TIA) due to the presence of a PFO, rather than with another cardiovascular condition, such as arrhythmias 4. Moreover, flecainide administration, sometimes used to treat atrial arrhythmias subsequent to PFO closure, has been demonstrated to be an unmasking factor for Brugada pattern on electrocardiogram (ECG)^5^.

## Case Report

**Figure 1:**
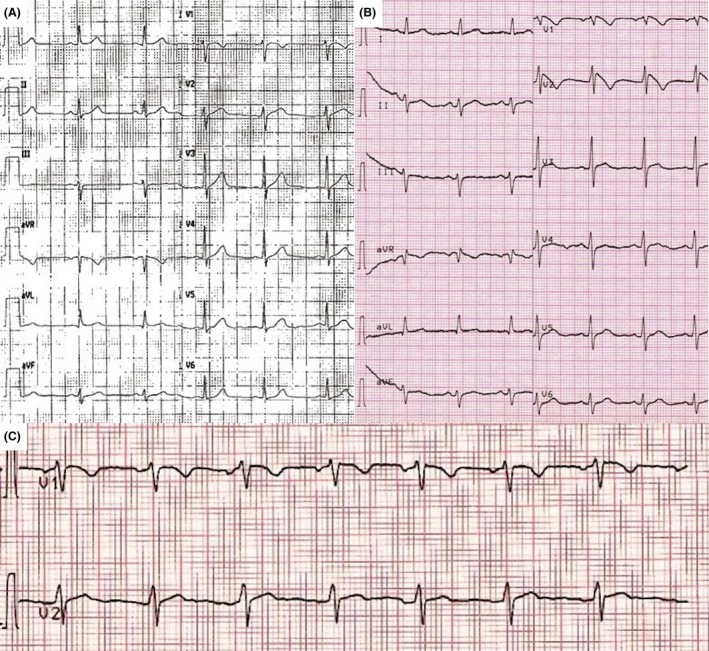
(A) Previous ECG, registered before PFO closure, appeared to be normal. (B) ECG performed in the emergency room of our hospital, showing type 1 Brugada pattern; the chronic oral intake of flecainide, prescribed because of supraventricular ectopic beats, likely unmasked the ECG pattern and contributed in symptoms worsening. (C) ECG normalization after flecainide discontinuation.

We report the case of a 58‐year‐old Caucasian female, admitted to our hospital for presyncope. Five years before, due to frequent episodes of migraine, presyncope, and paresthesia, she had undergone cerebral MRI with diffuse subcortical gliosis, ECG which appeared to be normal (Fig. 1A), and transthoracic echocardiography (TTE) showing an atrial septal aneurysm associated with a moderate right‐to‐left interatrial shunt, increased by Valsalva maneuver. As both contrast‐enhanced transcranial Doppler and transesophageal echocardiography had confirmed the presence of a PFO, the patient received device‐based percutaneous closure. After closure, she referred frequent palpitations and at that time ECG showed incomplete right bundle branch block and Holter monitoring revealed frequent supraventricular extrasystoles; so administration of oral flecainide therapy (100 mg twice daily) was started. Despite this therapeutic approach, she continued to refer symptoms due to palpitations, especially at night.

A further syncope with falling to the ground and consequent head injury led the patient to our observation. The ECG showed down sloping ST‐segment elevation in V1 and V2 leads, which was not evident in any previous ECGs (Fig. 1B).

Based on these findings and especially on the clinical history reported and the ECG morphology, diagnosis of type 1 Brugada syndrome (BrS) was made and the patient underwent implantable cardioverter defibrillator (ICD) implantation. After discontinuation of flecainide, an improvement of symptoms was referred and serial ECGs showed a progressive normalization of ST segment in V1‐V2 (Fig. 1C). The clinical follow‐up at 1 year showed absence of symptoms and ICD intervention.

## Discussion

The patient received PFO closure, in a first center, for symptoms attributable to transient cerebral ischemia and instrumental evidence of PFO and subcortical gliosis.

On the background of the clinical history of the patient, the morphological and functional characteristics of the PFO and the neuroimaging features of cerebral ischemia may provide useful information to understand the relationship between PFO and symptoms and the probability for PFO to be culprit or bystander 6, 7, 8. However, signs of subcortical gliosis are present in most people but often nonspecific, as well as the detection of a PFO is sometimes occasional and unrelated to the symptoms.

History of frequent supraventricular arrhythmias and palpitations has been described in the follow‐up of patients who underwent PFO closure 9. One of the most common antiarrhythmic drug, flecainide, is currently used for the diagnosis of Brugada syndrome, administered intravenously, under ECG monitoring 10, 11. In our case, flecainide administration was crucial to unmask a latent Brugada pattern on the ECG, leading, also based on the clinical history, to ICD implantation for prevention of malignant ventricular arrhythmias 12, 13, 14.

Considering this case presentation it is possible, in our opinion, that initial symptoms were not related to the presence of a PFO but already first manifestations of a BrS.

Another possible interpretation could be that the presyncope, before PFO closure, was really due to paradoxical embolization (as flecainide had not been administered at that time) leading to a proper PFO closure and then starting all the subsequent developments related to flecainide administration. In this perspective, the danger of improper flecainide prescription to patients with undiagnosed latent BrS needs to be pointed out.

## Conclusion

This report shows that careful evaluation of the clinical history and symptoms is extremely helpful for the correct diagnosis. It seems therefore evident that symptoms, presumed to be due to paradoxical embolism, may probably be caused by arrhythmic events related to the BrS; anyway, their following exacerbation can be triggered by an incautious flecainide administration after PFO closure. The presence of PFO is too often considered the first responsible for patient's symptoms when no other causes are apparently evident, leading to frequent misdiagnosis; however, a PFO is not always the guilty, but in many cases, it is just an innocent bystander.

## Conflict of Interest

None declared.

## Authorship

FV: involved in clinical care, decision making, and manuscript revision. GV: involved in writing and revision of the manuscript. DS, FR: revised the manuscript. GA: wrote and revised the manuscript. AT: involved in clinical care, decision making, and image preparation.
